# Distribution Patterns of Bitterness and Astringency Compounds in Different Tissues and Developmental Stages of Three Sympodial Bamboo Species

**DOI:** 10.3390/foods15050897

**Published:** 2026-03-05

**Authors:** Yuanyuan Li, Yilin Zheng, Xizhi Chen, Chang Xu, Huijuan Lu, Yangyang Zhang, Wentian Song, Xuejun Yu

**Affiliations:** 1 State Key Laboratory for Development and Utilization of Forest Food Resources, Zhejiang Agriculture and Forestry University, Hangzhou 311300, China; yuanli@zafu.edu.cn (Y.L.); yilinzheng_2001@foxmail.com (Y.Z.); xizhichen11@foxmail.com (X.C.); xuchang1999123@foxmail.com (C.X.); huijuanlu2001@foxmail.com (H.L.); zhangyangyang@zafu.edu.cn (Y.Z.); songwt@zafu.edu.cn (W.S.); 2 Bamboo Industry Institute, Zhejiang Agriculture and Forestry University, Hangzhou 311300, China; 3 Bamboo Shoot High-Efficiency Utilization Center, China National Academy of Bamboo Industry, Huzhou 313300, China

**Keywords:** bamboo shoots, bitter and astringent compounds, growth stages, descriptive sensory analysis, correlation

## Abstract

Bamboo shoots are valued as traditional vegetables, but their palatability is often compromised by bitter and astringent compounds. The spatial and temporal distribution of these compounds across species, tissues, and developmental stages remains poorly characterized. This study systematically investigated key taste-active compounds (tannins, oxalic acid, flavonoids, cyanide compounds, and free amino acids) in three sympodial bamboo species (*Bambusa chungii*, *Dendrocalamus farinosus*, and *Bambusa oldhamii*). We integrated quantitative chemical analysis of shoots at different emergence stages and tissue parts with descriptive sensory evaluation. The results revealed pronounced, species-specific accumulation patterns. For instance, tannin content increased with shoot emergence in all species, whereas oxalic acid and cyanide showed divergent temporal trends among them. Tissue-specific gradients were also evident for most compounds. Correlation analysis with sensory data indicated distinct associations for each species. Bitterness in *D. farinosus* was most strongly correlated with oxalic acid, while in *B. oldhamii*, it was closely linked to tannins and cyanide. In *B. chungii*, specific amino acids (aspartic acid, histidine) and tannins showed significant correlations with bitterness perception. The perception of astringency involved multiple contributing factors. These findings elucidate the distinct biochemical bases of flavor variation in sympodial bamboos. They provide a scientific rationale for optimizing harvest timing and tissue selection, offering targeted strategies for post-harvest processing to improve edible quality and market value.

## 1. Introduction

Bamboo shoots, the edible young culms of various bamboo species, have served as a staple food in many Asian cultures for centuries, with deep culinary roots in China, Japan, Korea, and India [1,2]. Among these, sympodial bamboos are particularly valued in agriculture for their rapid growth, high yield, and rich nutritional profile [1,3]. Widely cultivated genera for edible shoots include *Phyllostachys*, *Bambusa*, and *Dendrocalamus*, each adapted to distinct ecological niches and contributing to diverse regional dietary traditions [1,2]. Beyond their culinary appeal, bamboo shoots are a valuable source of dietary fiber, vitamins, minerals, and bioactive compounds. Their composition is primarily water (approximately 90%), accompanied by carbohydrates (including fiber), proteins, minerals such as potassium, phosphorus, and calcium, vitamins like vitamin C, and bioactive constituents including flavonoids and phenolic acids [4]. These components collectively underpin the nutritional and health-promoting properties of bamboo shoots.

Despite these benefits, the palatability of bamboo shoots is frequently limited by the presence of bitter and astringent compounds, the levels of which exhibit significant variation across species, developmental stages, and tissue types [5,6]. Bitterness, a taste modality mediated by TAS2R receptors, is commonly elicited in plant foods by secondary metabolites such as alkaloids, glycosides, terpenoids, and specific amino acids [7]. In bamboo shoots, bitterness is primarily attributed to an array of secondary metabolites including alkaloids, glycosides, flavonoids, cyanogenic compounds, and certain amino acids [5,8]. Recent advances in metabolomic and transcriptomic analyses have identified compounds like arbutin, phenylalanine, tryptophan, and various phenolic acids as key contributors to bitterness, with their synthesis and accumulation being closely tied to shoot emergence and exposure to light [5,8,9]. For example, the shift from belowground to aboveground growth is associated with a substantial increase in these bitter metabolites, especially in the apical shoot portions [5,8].

Astringency, another sensory attribute critical to quality, arises mainly from the interaction of salivary proteins with compounds such as condensed tannins (proanthocyanidins) and oxalic acid, resulting in a characteristic dry, puckering sensation [8]. The biosynthesis and accumulation of these polyphenolic compounds are influenced by a combination of environmental factors (e.g., light exposure, altitude, cultivation practices) and genetic differences among bamboo species [3,9]. Practical interventions, such as shading or soil-mulching, have been shown to suppress the formation of these undesirable compounds, thereby enhancing taste quality [9]. For instance, shading treatments reduce phenolic acids associated with the tyrosine pathway and decrease bitterness accumulation [10]. Conversely, factors like low temperatures can delay lignification and favor the formation of longer-chain tannin polymers [11]. Parallels can be drawn from viticulture, where excessive nitrogen fertilization reduces tannin content and subsequently alters wine astringency [12]. While a moderate level of astringency can be desirable, imparting a “dry, clean” palate-cleansing note in species like *Dendrocalamus latiflorus,* excessive tannin–protein interaction can lead to an unpleasant mouthfeel and potential digestive discomfort. Such negative effects can be mitigated through processing techniques like fermentation, boiling, or targeted reduction in tannin content [13].

The overall flavor profile of bamboo shoots is a complex balance of sweet, umami, bitter, and astringent notes. Sweetness and umami are derived mainly from soluble sugars and amino acids, whereas bitterness and astringency are linked to phenolic compounds, cyanogenic glycosides, and specific amino acids [5,8,9]. The dynamic spatial and temporal changes in these compounds during shoot development highlight the importance of understanding their distribution patterns to optimize harvest timing and post-harvest processing [5,8,9]. Although prior studies have identified several key bitter and astringent compounds in bamboo shoots, comprehensive analyses of their distribution across different species, developmental stages, and shoot tissues remain limited [5,8]. Moreover, many previous investigations have relied on metabolomic and transcriptomic data to associate upregulated metabolites with bitter taste; however, metabolite upregulation alone is insufficient to confirm sensory impact. Robust validation requires complementary sensory evaluation and dose-threshold analysis [5,8]. In this context, the commercially important sympodial bamboo species—*Bambusa oldhamii*, *Bambusa chungii*, and *Dendrocalamus farinosus*—which exhibit distinct gradients of bitterness and astringency, offer an ideal model system for elucidating the biochemical and sensory mechanisms underlying flavor variation [1,5,8].

In this study, we conducted a systematic evaluation of the content and distribution of the major compounds influencing bitter and astringent flavors in these three sympodial bamboo species across different growth stages and shoot tissues. By integrating quantitative chemical analysis with structured sensory evaluation, this research aims to clarify the specific compounds and their variation patterns responsible for bitterness and astringency, and to predict the optimal utilization period for each bamboo species. The findings are expected to provide a scientific basis for improving the edible quality and market value of bamboo shoots through targeted cultivation, harvesting, and processing strategies.

## 2. Materials and Methods

### 2.1. Plant Materials and Sample Preparation

Three different types of bamboo shoots were collected from Yibin, Sichuan Province, China. *Bambusa chungii* was harvested from the Century Bamboo Garden in Changning County, Yibin City (28°31′ N, 104°56′ E). *Dendrocalamus farinosus* was obtained from Renhe Town, Jiang’an County (28°30′ N, 105°9′ E). *Bambusa oldhamii* was collected from Song jia Town, Cui ping District (28°45′ N, 104°52′ E). All three locations are situated close to each other, with similar topography and minimal climate differences, falling within the subtropical humid monsoon climate zone and sharing characteristics of the South Asian tropical climate.

Bamboo shoots of *D. farinosus*, *B. oldhamii* and *B. chungii* were collected during the peak shoot emergence period, between June and October. All shoots of a given species were randomly sampled from the same bamboo forest at the same time. The shoots were categorized into three different growth stages based on the height of emergence: full emergence period (approximately 30 cm in height, with the above-ground part constituting 90% of the entire shoot), half emergence period (approximately 10 cm in height, with the below-ground part making up 50% of the shoot), and pre-emergence period (with a shoot length of about 10 cm). After sampling, each bamboo shoot was evenly divided into the tip, middle, and basal sections according to its length. One portion of each section was ground into a homogeneous pulp using a grinding mill and then frozen at −80 °C for future use.

### 2.2. Sensory Evaluation

The sensory evaluation experimental group consisted of 16 members, including researchers and graduate students from the Bamboo Research Institute of Zhejiang Agriculture and Forestry University. This panel size is in accordance with guidelines for descriptive analysis using trained panels as per referenced standards [14,15] and is comparable to panels employed in published sensory studies on tea and other vegetables with complex flavor profiles. Informed consent was obtained from all members involved in this study. The screening and training methods were based on the national standard [14,15]. Panelists were trained to recognize the minimum perception concentrations of the five basic tastes in aqueous solutions, including citric acid (1.2 g·L^−1^) for sour taste, folium llicis cournutae tea (0.54 g·L^−1^) for bitter taste, sodium chloride (4.0 g·L^−1^) for salty taste, sucrose (24.0 g·L^−1^) for sweet taste, and monosodium glutamate (2.0 g·L^−1^) for umami taste. For each flavor, 8 gradients of diluents were set in the order of increasing mass concentration, which were provided to sensory evaluators for different intensity recognition of the same taste.

The various samples of the three types of bamboo shoots were assigned random coded numbers. The experimental sensory evaluation personnel were limited to food and liquid consumption 2 h before the experiment and gargled with pure water before the sensory evaluation. During the evaluation, samples were kept in the mouth for 15~25 s and then chewed. When evaluating the taste intensity, first folium llici teas cournutae and zinc lactate were used in the known bitter and astringent standard solution as the reference standard (Table 1), and then the sensory evaluators assigned the taste intensity of the undetermined substance according to their strength level. Each evaluation was repeated three times.

### 2.3. Determination of Tannin Contents

Tannin content was determined by a spectrophotometric method [16]. Samples were taken as 2.0 g of bamboo shoot homogenate was mixed with 100 mL DI water and extracted in a 100 °C water bath for 40 min. Supernatant was obtained after centrifugation at 8000 *g* for 5 min. Then extracts were mixed with 5 mL of DI water, 1 mL of Folin–Denis reagent and 3.0 mL of Na_2_CO_3_ solution and were kept in the dark for 2 h to completely stabilize the color. The absorbance of sample solution and blank solution was determined at 765 nm. All assayed content tests were repeated three times.

### 2.4. Determination of Oxalic Acid Contents

The content of oxalic acid was determined by RP-HPLC [17]. Chromatographic conditions were as follows: mobile phase included 0.2% potassium dihydrogen phosphate and 0.3 mmol tetrabutylammonium hydrogen sulfate (pH = 2); column temperature was 30 °C, injection volume was 10 µL, flow rate was 1 mL/min. Weigh 5 g of fresh homogeneous bamboo shoots, place them in a 50.0 mL volumetric flask, dilute them to 40.0 mL with deionized water, and extract by ultrasonic wave for 1 h. After extraction and dilution to volume, take 2 mL of microporous filter membrane (water system, pore size 0.45 μm) for analysis.

### 2.5. Determination of Flavonoid Contents

A quantity of 1 g bamboo shoot homogenate sample was mixed with 50 mL of 70% ethanol and extracted with ultrasound. After centrifugation at 3000 *g* for 10 min, the supernatant was collected, and the residue was extracted twice using the same method. The combined extract was concentrated and then diluted to 100.0 mL with 70% ethanol. Slightly modified from Jia’s method [18], the absorbance was measured at 510 nm with rutin as the reference material. All assayed content tests were repeated three times.

### 2.6. Determination of Cyanide Contents

Cyanide content was quantified with minor modifications [19]. A sample size of 10 g bamboo shoot homogenate was mixed with 200 mL of water, 20 mL of 0.1 g/mL zinc acetate solution and 1 g of tartaric acid. The solution was then heated for distillation. Blank was prepared at the same time. Transfer 10.0 mL of equal distillate and equal blank distillate into a 25.0 mL colorimetric tube, add 1.0 g/L NaOH solution to 10.0 mL, and mix with 5.0 mL of phosphate buffer (pH = 7). After stirring, quickly add 0.2 mL of 10 g/L chloramine T solution, mix well, and stand for 3–5 min. Subsequently, 5.0 mL of pyrazolone nicotinate solution was added, diluted to the scale with water, and placed in a water bath at 25–35 °C for 40 min. Absorbance was measured at the wavelength of 638 nm. All assayed content tests were repeated three times.

### 2.7. Determination of Free Amino Acid Contents

Amino acids were extracted by acid hydrolysis [20]. Weigh 0.5 g of bamboo shoot homogenate mixed with 10.0 mL of 6 mol/L hydrochloric acid and hydrolyzed in an oven at 110 °C for 24 h. After cooling, samples were diluted, then dried with a nitrogen blower, and dissolved in 1.0 mL of 0.02 mol/L hydrochloric acid. Then, full-automatic amino acid analyzer was used for detection and analysis. The amino acids were quantified as: umami (Asp + Glu), bitter (Val + Ile + Leu + Tyr + Phe + Tyr), sweet (Gly + Thr + Ala + Ser) and essential amino acid (Thr + Val + Ile + Leu + Lys + Met + Cys + Phe + Tyr) [20].

### 2.8. Data Analysis

All data were statistically analyzed using one-way ANOVA in SPSS Statistics 22.0, followed by multiple comparison tests. Origin 2021 was used for drawing figures. Simca 14.1 was used for OPLS analysis.

## 3. Results

### 3.1. Tannin Content in Different Bamboo Shoot Varieties, Growing Stages and Tissue Parts

**Figure 1:**
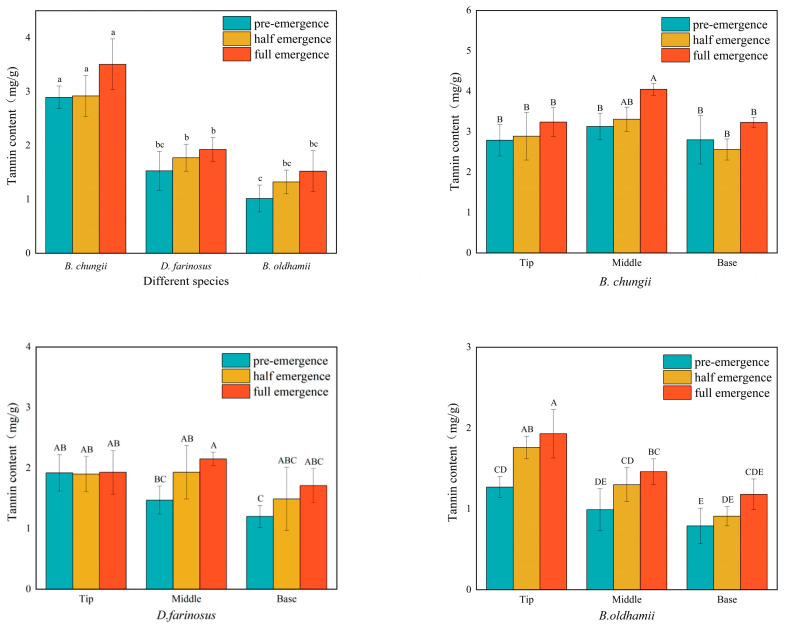
Tannin content. *B. chungii* refers to *Bambusa chungii*, *D. farinosus* to *Dendrocalamus farinosus*, and *B. oldhamii* to *Bambusa oldhamii*. Different lowercase letters indicate significant differences among different bamboo species (*p* < 0.05), and different uppercase letters indicate significant differences within the same bamboo species (*p* < 0.5). The same applies for figures below.

As shown in Figure 1, the total tannin content in all three bamboo shoot varieties increased with growth time. *B. chungii* reached its peak total tannin content during the full emergence period at 3.51 mg/g, while *D. farinosus* and *B. oldhamii* had contents of 1.93 mg/g and 1.52 mg/g, respectively. The tannin content was lowest in the base portion of all three bamboo shoots, with *D. farinosus* and *B. oldhamii* having the lowest contents when unemerged (1.20 mg/g and 0.79 mg/g, respectively), while *B. chungii* had the lowest content during partial emergence at 2.56 mg/g.

### 3.2. Oxalic Acid Content in Different Bamboo Shoot Varieties, Growing Stages and Tissue Parts

**Figure 2:**
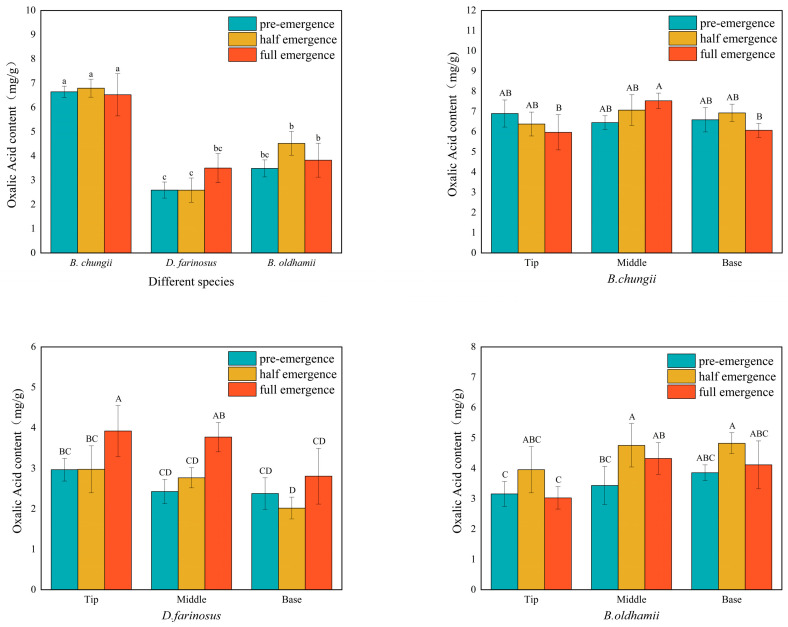
Oxalic acid content.

As shown in Figure 2, the total oxalic acid content in *B. chungii* remained relatively stable over time, ranging from 6.52 to 6.80 mg/g, with no significant differences between different portions. The total oxalic acid content in *D. farinosus* increased with time, reaching its peak during the full emergence period at 3.50 mg/g, with the tip portion having the highest content. The total oxalic acid content in *B. oldhamii* first increased and then decreased with time, reaching its peak during partial emergence at 4.52 mg/g, with the base portion having the highest content.

### 3.3. Flavonoid Content in Different Bamboo Shoot Varieties, Growing Stages and Tissue Parts

**Figure 3:**
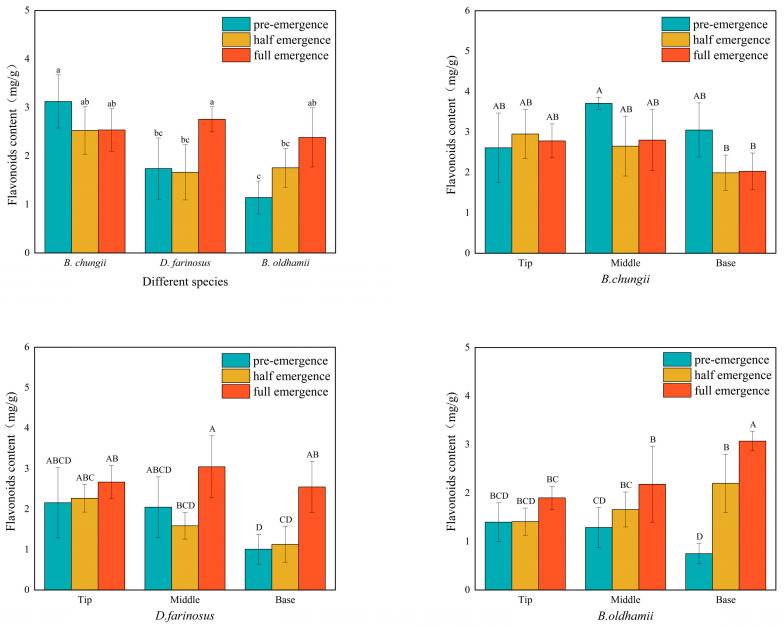
Flavonoid content.

As shown in Figure 3, the total flavonoid content in *B. chungii* reached its peak during the unemerged period at 3.12 mg/g; then, it decreased and subsequently increased with growth time, but overall changes were minimal. The middle portion had the highest content during the unemerged period. The total flavonoid content in *D. farinosus* and *B. oldhamii* both reached their peaks during the full emergence period at 2.76 mg/g and 2.38 mg/g, respectively. Both had the lowest content in the base portion when unemerged. However, the total flavonoid content in *D. farinosus* first decreased and then increased with growth time, while that in *B. oldhamii* increased with growth time.

### 3.4. Cyanide Content in Different Bamboo Shoot Varieties, Growing Stages and Tissue Parts

**Figure 4:**
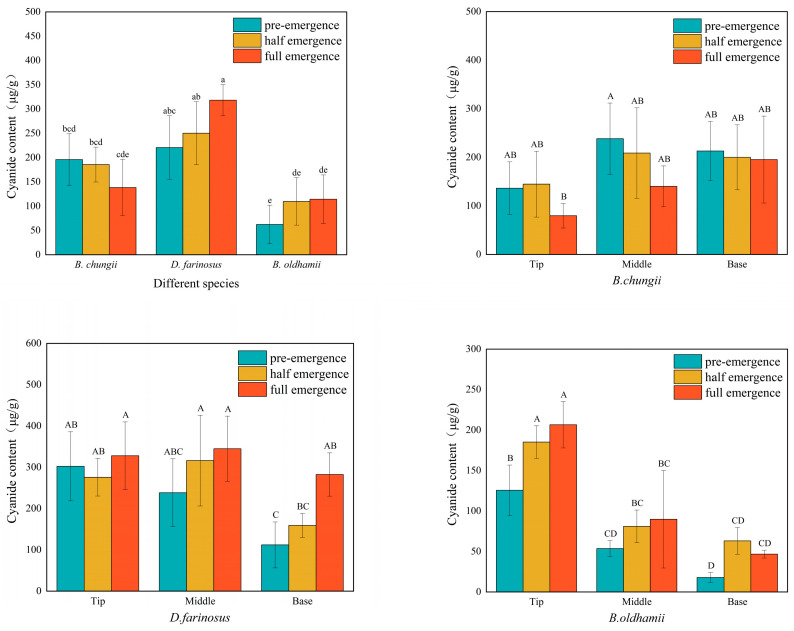
Cyanide content.

As shown in Figure 4, the total cyanide content in *B. chungii* reached its peak during the unemerged period at 196.03 μg/g and then decreased with time. The middle portion had the highest content when unemerged. The total cyanide content in *D. farinosus* and *B. oldhamii* both increased with growth time, reaching their peaks during the full emergence period at 318.29 μg/g and 114.38 μg/g, respectively. The base portion had the lowest content when unemerged.

### 3.5. Amino Acid Content in Different Bamboo Shoot Varieties, Growing Stages and Tissue Parts

**Figure 5:**
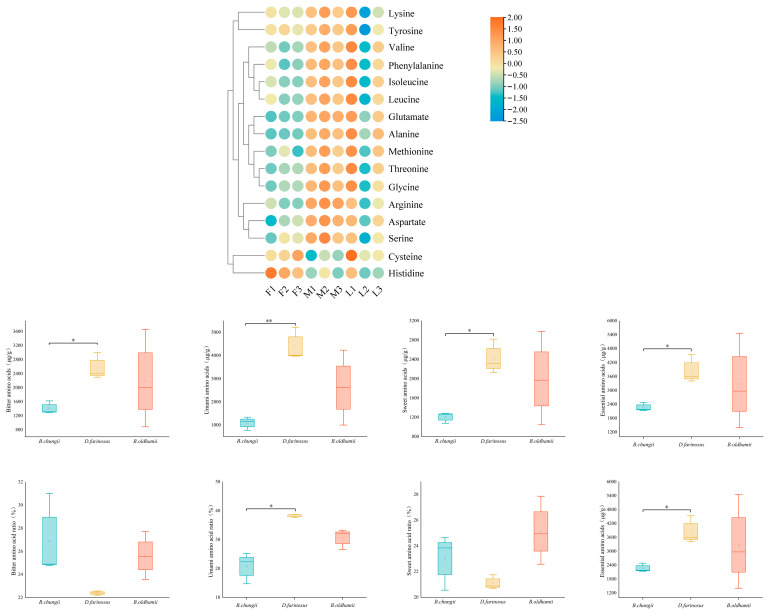
Changes in amino acid content. “F” refers to *Bambusa chungii*, “M” to *Dendrocalamus farinosus*, and “L” to *Bambusa oldhamii*. The numbers 1, 2, 3 in the heatmap correspond to full emergence, half emergence, and pre-emergence, respectively. An asterisk (*) and double asterisks (**) denote significant differences between species at *p* < 0.05 and *p* < 0.01, respectively. Umami (Asp + Glu), bitter (Val + Ile + Leu + Tyr + Phe + Tyr), sweet (Gly + Thr + Ala + Ser) and essential amino acid (Thr + Val + Ile + Leu + Lys + Met + Cys + Phe + Tyr). The proportions of umami, bitter, sweet and essential amino acids are calculated as their respective percentages of the total amino acid content [20].

The amino acid content differed among bamboo species. The heatmap in Figure 5 showed that the total amino-acid level in *D. farinosus* was markedly higher than in the other two species. Specifically, after grouping all amino acids, the analysis revealed that the content of bitter, umami, sweet and essential amino acids in *D. farinosus* is significantly more abundant than in the other species (Figure 5), reaching an average of 2567.37 μg/g, 4398.73 μg/g, 2419.27 μg/g and 3847.67 μg/g, respectively. While the contents of bitter and essential amino acids were the highest when *B. oldhamii* was fully unearthed, they can reach 3660.70 μg/g and 5454.90 μg/g, respectively.

### 3.6. Sensory Evaluation in Bitter and Astringent Taste in Three Bamboo Varieties

As shown in Table 2, the sensory scores varied among the three bamboo species. The bitter compounds in three bamboo species differ, as shown in Table 3. According to Pearson correlation analysis, the main bitter compounds in *B. chungii* are tannins, aspartic acid and histidine. The primary bitter compound in *D. farinosus* is oxalic acid. The main bitter compounds in *B. oldhamii* are tannins and cyanide. The order of VIP value greater than 1 is cyanide, oxalic acid, and tannin (Section 3.7). The astringency of all three bamboo species was significantly influenced by cyanide, oxalic acid, and tannin. As shown in Table 3, according to Pearson correlation analysis, the astringent-contributing substances in *B. chungii* and *D. farinosus* were not significant, while cyanide was the primary astringent substance in *B. oldhamii*. The expression of bitter and astringent substances in bamboo shoots was influenced by multiple factors. Major factors correlated to astringent taste in *B. chungii* and *D. farinosus* bamboo shoots were not denoted. Future analysis specifically separating these factors by gradients is needed to elucidate the mechanism.

### 3.7. OPLS-DA Analysis for Contributors in Bitter and Astringent Taste in Three Bamboo Varieties

The contribution of different indexes to the bitterness and astringency values was studied, and the sensory values of bitterness and astringency were taken as dependent variables (Y), and taste contributors, such as oxalic acid and flavonoids, were taken as independent variables (X). The OPLS-DA method distinguished the bitterness and astringency of three distinct bamboo species. Studies indicated that, while the three bamboo species can be distinguished to some extent, their correlations were not significant, or no primary influencing factor has been identified, suggesting that sensory perception is a complex process involving multiple taste-related factors. At high concentrations, sweetness may be masked by bitterness, as if a food contains both bitter and sweet flavors; the stronger the sweetness, the more likely the bitterness was to be perceived, creating a ‘sweet and bitter’ perceptual illusion [21]. For instance, monosaccharides can attenuate bitterness by blocking bitter taste receptors [22]. Other studies have demonstrated that the sour taste is perceived as dominant, leading to a ‘transfer of dominance,’ which paradoxically suppresses the bitter taste [23]. It is challenging to establish definitive correlations across different bamboo species or parts, further demonstrating the limitations and unreliable conclusions of previous reports that identified upregulated key components from metabolomics and transcriptomics as primary bitter compounds. Future research could explore the contributing factors of low-dose high bitterness/stringiness by controlling multiple taste factors and selectively regulating specific bitter substances. Only when the content change in a certain substance is highly correlated with the intensity of sensory bitterness, and it is verified through pure product that it can indeed cause bitterness, can it be identified as a key bitter substance.

**Figure 6:**
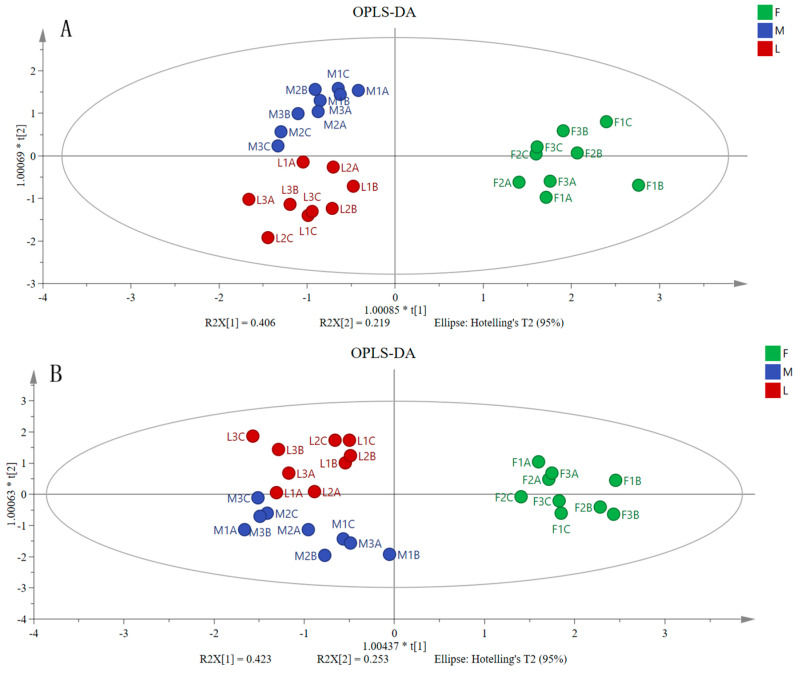
OPLS-DA analysis of bitterness (**A**) and astringency (**B**) of three bamboo species. “F” refers to *Bambusa chungii*, “M” to *Dendrocalamus farinosus*, and “L” to *Bambusa oldhamii*. 1, 2, and 3 correspond to full emergence, half emergence, and pre-emergence, respectively. A, B, and C correspond to the tip, middle, and base sections, respectively. The asterisk (*) in the axis labels indicates a scaling factor applied to the principal component scores (t[1] and t[2]) for visualization, which does not affect the model interpretation.

OPLS-DA was employed as an exploratory tool to visualize whether the measured chemical profiles could differentiate samples based on species and sensory attributes (Figure 6). It is important to note that this multivariate analysis suggests potential associations, not causal links, and its interpretation is constrained by the variables included in the model.

## 4. Discussion

### 4.1. Overview of Taste-Active Compounds in Bamboo Shoots

Bitterness, a fundamental taste mediated by T2R receptors, critically influences food palatability [24,25]. In bamboo shoots, this sensation and astringency are primarily attributed to a suite of compounds including tannins [26], cyanogenic compounds [27], free amino acids [8,17,28], flavonoids [29], and oxalic acid. Our integrated chemical and correlation analyses reveal that the key drivers of bitterness perception are distinct and highly species-specific among the three sympodial bamboos studied.

### 4.2. Species-Specific Roles of Major Compounds

#### 4.2.1. Tannins and Cyanide in *Bambusa oldhamii*

Bitterness in *B. oldhamii* was predominantly associated with tannins and cyanide. Tannins, polyphenolics known for contributing to bitterness and astringency in foods like wine and tea [30,31,32], increased with growth duration in all species, aligning with known light-induced biosynthesis [5]. The significant correlation in *B. oldhamii*, however, underscores its particular sensitivity. Cyanide content also increased over time in this species, contrasting with its decrease in *B. chungii*. This pattern, potentially linked to light exposure as suggested by studies on husk color [33], indicates cyanogenic glycosides as a co-contributor to its bitter profile.

#### 4.2.2. Oxalic Acid in *Dendrocalamus farinosus*

In *D. farinosus*, oxalic acid emerged as the compound most strongly correlated with bitterness. Oxalic acid’s primary sensory role is often sour/astringent, and its strong correlation in our model may reflect a combined sensory impact or co-occurrence with other compounds. Its significant correlation with the overall undesirable taste (bitterness/astringency) might be a result of the intensified perception of bitterness caused by the overall outcome, such as combining the taste and feeling caused by the denaturation of salivary protein. While commonly associated with sourness and astringency [34,35], oxalic acid’s primary role here is identified as a major driver of undesirable taste in this species. Its content increased consistently over time, a trend distinct from the other species and possibly regulated by factors such as temperature or water availability beyond just light [36], highlighting unique metabolic regulation.

#### 4.2.3. Complex Interactions in *Bambusa chungii*

*B. chungii* presented a more complex pattern, with significant correlations for tannins and specific amino acids (aspartic acid and histidine). The involvement of aspartic acid, a primary umami amino acid, suggests a nuanced taste interaction where these compounds may influence bitterness within the specific concentration matrix of this species. This unique profile is further evidenced by its distinct metabolic dynamics, such as decreasing cyanide and stable oxalic acid content post-emergence [36].

### 4.3. Other Compounds and Implications

#### 4.3.1. Flavonoids

Although flavonoids are recognized as bitter/astringent agents in plants like citrus and tea [37,38] and show light-dependent fluctuations in bamboo shoots [39,40], they did not emerge as a primary correlative factor for bitterness in this study, suggesting that their contribution may be secondary or synergistic under these conditions.

#### 4.3.2. General Biochemical Pathways

The species-dependent accumulation of these compounds likely stems from genetic differences governing divergent secondary metabolic pathways [1]. For instance, the synthesis of oxalic acid is regulated by multiple environmental factors [36], while cyanide and flavonoid levels are significantly influenced by light exposure [33,40].

### 4.4. Implications for Harvest and Processing

The biochemical basis of bitterness is thus species-specific. Consequently, management strategies must be tailored. Harvesting at the pre-emergence stage generally minimizes bitterness. Post-harvest interventions like light shielding [10,41], microbial fermentation [27,42], or adjusted nitrogen deposition [43] should be optimized based on the target species’ dominant bitter compound profile (e.g., focusing on tannin reduction for *B. oldhamii* versus oxalic acid control for *D. farinosus*) to most effectively enhance palatability.

### 4.5. Limitations and Future Perspectives

First, while the sensory panel was trained, its size was moderate. Studies involving larger consumer panels could further validate the sensory correlations. Additionally, the use of total amino acid analysis, while informative for correlative screening, does not differentiate free from bound forms. Future studies employing free amino acid-specific profiling could refine our understanding of the direct taste-active compounds. Second, the multivariate models (e.g., OPLS-DA) used are exploratory, and their outcomes are influenced by variable selection. Future studies should include more sound analysis such as ANNs and other models to further elucidate the subject. Finally, this work quantified compound groups rather than specific molecules. Future research identifying specific molecular entities, such as individual tannin polymers, would offer deeper mechanistic insight.

## 5. Conclusions

The key aspect of this study was the use of a combination of chemical and sensory evaluations to examine the distribution of bitter compounds in different periods and locations. It was found that there were significant differences in the content of bitter and astringent substances among the three types of bamboo studied and different growth stages. Oxalic acid compounds were identified as major factors correlating to bitterness and astringency in *D. farinosus*, while tannins and cyanide predominated in *B. oldhamii*, and specific amino acids (aspartic acid, histidine) and tannins were key in *B. chungii*. Univariate correlation analysis identified species-specific key correlative compounds, while the multivariate model illustrated the complexity and lack of a universal single-factor model. Thus, the “key contributor” claims are species-specific and based on correlation strength, not an absolute biological dominance. Such differences may be attributable to genetic factors, the influence of growth environment on metabolism and its products, variations in secondary metabolic pathways, or the production of different chemical defense substances as a result of ecological adaptations. These findings lay the foundation for further isolation and analysis of the key bitter compounds present in bamboo shoots. Future research could incorporate intelligent sensory technologies such as electronic tongues to more objectively and accurately quantify the bitter taste of bamboo shoots, thereby obtaining reliable and reproducible data.

To summarize, different species of bamboo possess the best taste and nutritional value before being harvested from the ground; at this stage, the bamboo shoots are mild in bitterness and rich in nutrients. Therefore, it is crucial to scientifically manage the timing of harvesting. The research findings provide insights that can help optimize cultivation, harvesting, and processing practices in order to improve the taste and quality of bamboo shoots. Future studies should focus on validating the direct causal role of these key compounds through dose–response assays and receptor studies, and developing targeted processing technologies to modulate their levels.

## Figures and Tables

**Table 1:** Criteria for sensory evaluation of bitter and astringency.

Concentration of Bitter Folium Llicis Cournutaetea/(mg·L^−1^)	Bitterness Value	Concentration of Zinc Lactate/(mg·L^−1^)	Astringency Value
300	10	1500	10
200	8	1000	8
150	6	700	6
100	4	400	4
25	2	150	2
0	0	0	0

**Table 2:** Sensory evaluation of three bamboo species.

Bamboo Species	Index	Full Emergence			Half Emergence			Pre-Emergence		
		Tip	Middle	Base	Tip	Middle	Base	Tip	Middle	Base
*B. chungii*	Bitterness	3.00 ± 0.60 b	4.86 ± 1.26 a	1.79 ± 0.59 de	2.29 ± 0.82 cd	2.50 ± 0.60 bc	2.42 ± 0.70 bcd	2.00 ± 0.55 cde	1.50 ± 0.39 e	2.21 ± 0.59 cd
	Astringency	3.54 ± 0.44 c	5.29 ± 0.58 a	1.29 ± 0.29 g	3.71 ± 0.49 c	4.15 ± 0.58 b	3.02 ± 0.30 d	2.71 ± 0.45 de	2.01 ± 0.16 f	2.57 ± 0.53 e
*D. farinosus*	Bitterness	9.64 ± 0.38 a	4.00 ± 0.54 b	2.29 ± 0.65 d	4.50 ± 1.21 b	3.00 ± 0.73 c	2.00 ± 0.75 de	2.57 ± 0.62 cd	3.95 ± 0.54 b	1.48 ± 0.41 e
	Astringency	3.00 ± 0.55 de	5.29 ± 0.71 a	1.31 ± 0.33 g	3.64 ± 0.52 c	4.14 ± 0.51 b	3.05 ± 0.26 d	2.79 ± 0.27 de	2.00 ± 0.54 f	2.57 ± 0.46 e
*B. oldhamii*	Bitterness	5.38 ± 0.48 a	2.40 ± 0.42 c	1.60 ± 0.50 de	3.81 ± 0.57 b	1.57 ± 0.36 de	1.40 ± 0.46 de	1.69 ± 0.40 d	1.19 ± 0.34 e	1.59 ± 0.36 de
	Astringency	5.02 ± 0.50 ab	2.41 ± 0.41 d	2.21 ± 0.30 de	4.79 ± 0.62 b	3.86 ± 0.54 c	4.81 ± 0.69 b	5.31 ± 0.48 a	2.62 ± 0.37 d	1.81 ± 0.45 e

Different lowercase letters indicate significant differences within the same bamboo species (*p* < 0.5).

**Table 3:** Pearson correlation analysis of three bamboo species.

Bamboo Species	Index	Tannin	Oxalic Acid	Flavonoids	Cyanide	Aspartate	Histidine
*B. chungii*	Bitterness	0.739 *	0.577	−0.076	−0.490	−0.998 *	1.000 **
	Astringency	0.552	0.647	0.071	−0.435	−0.583	0.629
*D. farinosus*	Bitterness	0.440	0.745 *	0.557	0.560	−0.220	−0.197
	Astringency	0.624	0.461	0.222	0.458	0.858	0.870
*B. oldhamii*	Bitterness	0.894 **	−0.437	0.022	0.863 **	0.444	0.822
	Astringency	0.502	−0.194	−0.035	0.682 *	−0.922	−0.620

* *p* < 0.05, ** *p* < 0.01.

## Data Availability

The original contributions presented in the study are included in the article, further inquiries can be directed to the corresponding author.
